# A population-based cohort study of mortality of intensive care unit patients with liver cirrhosis

**DOI:** 10.1186/s12876-020-1163-1

**Published:** 2020-01-16

**Authors:** Yu-Feng Huang, Chao-Shun Lin, Yih-Giun Cherng, Chun-Chieh Yeh, Ray-Jade Chen, Ta-Liang Chen, Chien-Chang Liao

**Affiliations:** 1 Department of Anesthesiology, Taitung MacKay Memorial Hospital, Taitung, Taiwan; 2 Department of Anesthesiology, Shuang Ho Hospital, Taipei Medical University, New Taipei City, Taiwan; 3 Department of Anesthesiology, School of Medicine, College of Medicine, Taipei Medical University, Taipei, Taiwan; 4 Department of Anesthesiology, Taipei Medical University Hospital, Taipei, Taiwan; 5 Anesthesiology and Health Policy Research Center, Taipei Medical University Hospital, Taipei, Taiwan; 6 Department of Surgery, China Medical University Hospital, Taichung, Taiwan; 7 Department of Surgery, University of Illinois, Chicago, USA; 8 Department of Surgery, Taipei Medical University Hospital, Taipei, Taiwan; 9 Department of Surgery, School of Medicine, College of Medicine, Taipei Medical University, Taipei, Taiwan; 10 Department of Anesthesiology, Wan Fang Hospital, Taipei Medical University, Taipei, Taiwan; 11 Research Center of Big Data and Meta-Analysis, Wan Fang Hospital, Taipei Medical University, Taipei, Taiwan; 12 School of Chinese Medicine, College of Chinese Medicine, China Medical University, Taichung, Taiwan

**Keywords:** Intensive care unit, Liver cirrhosis, Mortality

## Abstract

**Background:**

The impact of liver cirrhosis on the outcomes of admission to intensive care unit (ICU) is not completely understood. Our purpose is to identify risk factors for mortality in ICU patients with liver cirrhosis.

**Methods:**

Using reimbursement claims from Taiwan’s National Health Insurance Research Database from in 2006–2012, 1,250,300 patients were identified as having ICU stays of more than 1 day, and 37,197 of these had liver cirrhosis. With propensity score-matching for socioeconomic status, pre-existing medical conditions, and cirrhosis-related morbidities, 37,197 ICU patients without liver cirrhosis were selected for comparison. Adjusted odds ratios (ORs) and 95% confidence intervals (CIs) of cirrhosis associated with 30-day, ICU, and one-year mortality were calculated.

**Results:**

Compared with control, cirrhotic patients had higher 30-day mortality (aOR 1.60, 95% CI 1.53 to 1.68), particularly those with jaundice (aOR 2.23, 95% CI 2.03 to 2.45), ascites (aOR 2.32, 95% CI 2.19 to 2.46) or hepatic coma (aOR 2.21, 95% CI 2.07 to 2.36). Among ICU patients, liver cirrhosis was also associated with ICU mortality (aOR 144, 95% CI 1.38 to 1.51) and one-year mortality (aOR 1.40, 95% CI 1.35 to 1.46). Associations between cirrhosis of liver and increased 30-day mortality were significant in both sexes and every age group.

**Conclusions:**

Liver cirrhosis was associated with 30-day mortality in ICU patients. Jaundice, ascites, hepatic coma, more than 4 admissions due to cirrhosis, and more than 30 days of hospital stay due to cirrhosis were exacerbated factors in cirrhotic ICU patients.

## Background

Cirrhosis of the liver results in various complications and mortality worldwide, but especially in developed regions [1]. It is the fourth most common cause of death in Europe and leads to more than a million deaths around the globe annually [2, 3]. The main etiologies of liver cirrhosis in most areas are infection with hepatitis B or C virus and alcohol abuse [1]. A French screening program estimated prevalence at 0.3%, and European studies found annual incidences of 15.3–132.6 per 100,000 people [3].

In the United States, annual medical expenditures related to intensive care unit (ICU) admissions are US $3 billion, with mean charges of US $116,200 per admission [4]. Patients in late-stage liver cirrhosis are likely to be admitted to ICUs for critical conditions such as sepsis and renal or respiratory failure [4, 5]. Though some studies reported improving outcomes in patients with cirrhosis admitted to ICUs [6], the prognosis remains poor, with mortality rates as high as 45% or even higher [7, 8].

It is important to identify the risk factors of cirrhosis of the liver and associated adverse outcomes of patients admitted to ICU. Several prognostic scoring systems have been proposed for risk assessment [9–12], such as the Child-Pugh score [13], the Model for End-stage Liver Disease [14], the Acute Physiology and Chronic Health Evaluation [15], and the Sequential Organ Failure Assessment [16]. Most previous reports only analyzed risk factors that predict outcomes when stratifying cirrhotic patients admitted to ICU, but did not assess the influence of cirrhosis itself on mortality in ICU or one-year survival after discharge.

We conducted a nationwide population-based retrospective cohort study using Taiwan’s National Health Insurance Research Database to investigate ICU mortality in patients with and without liver cirrhosis. We also evaluated the impacts of different comorbidities and cirrhosis-related clinical indictors on ICU mortality and on one-year survival in further stratified analyses.

## Methods

### Data sources

We conducted this study using reimbursement claims data from Taiwan’s National Health Insurance Program. This program merged former insurance systems in March 1995 and covers more than 99% of Taiwan’s 23 million residents. The National Health Research Institutes established a National Health Insurance Research Database (NHIRD) to record all beneficiaries’ inpatient and outpatient medical services. This information includes basic patient demographics, physician’s primary and secondary disease diagnoses, treatment procedures, prescribed medications and medical expenditures for all health care services. The validity of this database has been favorably evaluated, and research articles based on it have been accepted in prominent scientific journals worldwide [17–20].

### Ethics

This study was conducted in accordance with the Helsinki Declaration. To protect personal privacy, the electronic database was decoded with patient identifications scrambled for further academic access for research. According to Taiwan National Health Research Institutes regulations, informed consent is not required because patient identifications were decoded and scrambled [18–20]. Ethical approval for this study (TMU-JIRB-201504008) was provided by the Institutional Review Board of Taipei Medical University.

### Study design

Among 23 million beneficiaries, 1,250,300 patients were admitted to ICU between 2006 and 2012 (Additional file 1: Figure S1). We identified 79,528 patients aged ≥20 years who had histories of liver cirrhosis from the National Health Insurance Research Database. Patients with liver cirrhosis were defined as having at least two visits for medical care with physician’s primary diagnosis of liver cirrhosis within the 24 months before ICU admission. To select appropriate comparison groups, we matched each ICU patient with cirrhosis with one randomly selected ICU patients without liver cirrhosis by the analysis with a propensity score-matched pair procedure (case-control ratio = 1:1). These matched factors included age, sex, low income, stay in medical center or not, diabetes, hypertension, mental disorder, chronic obstructive pulmonary disease, fracture, pneumonia, stroke, asthma, traumatic brain injury, congestive heart failure, immune thrombocytopenia, renal dialysis, hyperlipidemia, epilepsy, atrial fibrillation, peripheral vascular disease and systemic lupus erythematosus, causes of admission to ICU according to physician’s primary diagnosis (digestive disease, cancer, respiratory disease, circulatory disease, infectious disease, injury and poisoning, symptom-defined conditions, genitourinary disease, endocrine disease, musculoskeletal disease, neurological disease, skin disease, mental disorder, tumors, blood diseases, congenital anomalies, disease of perinatal period, complications of pregnancy, ICU complications (such as septicemia, pneumonia, acute renal failure, urinary tract infection, stroke, acute myocardial infarction and pulmonary embolism). After matching selection, there were 37,197 patients with cirrhosis of liver in the exposure group and 37,197 people without liver cirrhosis in non-exposure group. We investigated the impact of liver cirrhosis on 30-day mortality, ICU mortality, and one-year mortality among ICU patients in this study.

### Measures and definitions

Income status was identified by defining low-income patients as those who qualified for waived medical copayment, as this status is verified by the National Health Insurance Bureau. Whether patients stayed in medical center ICUs or those in other hospitals was also recorded. We used the *International Classification of Diseases, Ninth Revision, Clinical Modification* (ICD-9-CM) to define coexisting medical conditions and ICU complications. Details codes of ICD-9-CM for these diseases were listed in Additional file 1: Table S1. Cirrhosis of liver before ICU stay was defined as the major exposure. Coexisting medical conditions determined from medical claims within the 24-month period before ICU stay included diabetes, hypertension, mental disorders, chronic obstructive pulmonary disease, fracture, pneumonia, stroke, asthma, traumatic brain injury, congestive heart failure, immune thrombocytopenia, hyperlipidemia, epilepsy, atrial fibrillation, peripheral vascular disease, and systemic lupus erythematosus. Renal dialysis was defined by administration code (D8, D9). Seven major complications during the ICU stay were analyzed (and those having severe cases of these diseases before ICU were excluded) including septicemia, pneumonia, acute renal failure, urinary tract infection, stroke, acute myocardial infarction, and pulmonary embolism. Length of hospital stay and ICU medical expenditure were analyzed as secondary outcomes.

Causes of admission to ICU (according to physician’s primary diagnosis at admission) were also identified and described with disease codes including digestive disease, cancer, respiratory disease, circulatory disease, infectious disease, injury and poisoning, symptom-defined conditions, genitourinary disease, endocrine disease, musculoskeletal disease, neurological disease, skin disease, mental disorder, tumor, blood disease, congenital anomalies, disease of perinatal period, and pregnancy complications.

### Statistical analysis

To reduce confounding bias, we used a propensity score-matched pair combined with frequency matching procedure to balance the covariates between ICU patients with and without liver cirrhosis. We developed a non-parsimonious multivariable logistic regression model to estimate a propensity score for pre-ICU cirrhosis of the liver. We matched cirrhotic patients to patients without liver cirrhosis, using a greedy matching algorithm (without replacement) with a caliper width of 0.2 SDs of the log odds of the estimated propensity score. Clinical significance guided initial choices of covariates in this multivariable logistic regression model: age, sex, low income, ICU stay in medical center or not, diabetes, hypertension, mental disorders, chronic obstructive pulmonary disease, fracture, pneumonia, stroke, asthma, traumatic brain injury, congestive heart failure, immune thrombocytopenia, renal dialysis, hyperlipidemia, epilepsy, atrial fibrillation, peripheral vascular disease, systemic lupus erythematosus, septicemia, pneumonia, acute renal failure, urinary tract infection, stroke, acute myocardial infarction, pulmonary embolism, digestive disease, cancer, respiratory disease, circulatory disease, infectious disease, injury and poisoning, symptom-defined conditions, genitourinary disease, endocrine disease, musculoskeletal disease, neurological disease, skin disease, mental disorder, tumor, blood disease, congenital anomalies, disease of perinatal period, and complications of pregnancy. A structured iterative approach was used to refine this model to achieve covariate balance within matched pairs. We used chi-square tests to measure covariate balance, and *p* < 0.05 was suggested to represent meaningful covariate imbalance. We matched patients with and without cirrhosis using a greedy-matching algorithm with a caliper width of 0.2 SD of the log odds of the estimated propensity score. This method could remove 98% of bias from measured covariates.

Adjusted odds ratios (aORs) with 95% confidence intervals (CIs) for 30-day mortality, ICU mortality, and one-year mortality for patients with and without cirrhosis were analyzed with multiple logistic regression models by controlling for age, sex, low income, stay in medical center or not, coexisting medical conditions, ICU complications and admission causes. To confirm associations between liver cirrhosis and ICU mortality, we also performed stratification analysis by age, sex, low income, stay in medical center or not, coexisting medical conditions, ICU complications and causes of ICU admission. The impacts of liver-related indicators and medical care on 30-day mortality in ICU patients with cirrhosis were also measured by calculating adjusted ORs and 95% CIs in the multivariate logistic regression models. SAS version 9.1 (SAS Institute Inc., Cary, NC, USA) statistical software was used for data analyses; two-sided *p* < 0.05 indicated significant differences.

## Results

The Additional file 1: Figure S1 shows the flow chart for selecting ICU patients with and without liver cirrhosis. Table 1 shows the distributions of age, sex, low income, stay in medical center or not, coexisting medical conditions (diabetes, hypertension, mental disorders, chronic obstructive pulmonary disease, fracture, pneumonia, stroke, asthma, traumatic brain injury, congestive heart failure, idiopathic thrombocytopenic purpura, renal dialysis, hyperlipidemia, epilepsy, atrial fibrillation, peripheral vascular disease, systemic lupus erythematosus), ICU complications (septicemia, pneumonia, acute renal failure, urinary tract infection, stroke, acute myocardial infarction, pulmonary embolism), causes of admission to ICU (digestive disease, cancer, respiratory disease, circulatory disease, infectious disease, injury and poisoning, symptom-defined conditions, genitourinary disease, endocrine disease, musculoskeletal disease, neurological disease, skin disease, mental disorders, tumor, blood disease, congenital anomalies, disease of perinatal period, pregnancy complications) as well as surgery and endotracheal intubation balanced between surgical patients with and without cirrhosis of liver using the matching procedure by propensity score.

**Table 1:** Characteristics of ICU patients with and without liver cirrhosis

Pre-hospital and in-hospital characteristics	No liver cirrhosis (*N* = 37197)		Liver cirrhosis (*N* = 37197)	
Age, years	n	(%)	n	(%)
20–29	304	(0.8)	304	(0.8)
30–39	2261	(6.1)	2261	(6.1)
40–49	6022	(16.2)	6022	(16.2)
50–59	8939	(24.0)	8939	(24.0)
60–69	7832	(21.1)	7832	(21.1)
70–79	7841	(21.1)	7841	(21.1)
≥ 80	3998	(10.8)	3998	(10.8)
Sex				
Female	10,274	(27.6)	10,274	(27.6)
Male	26,923	(72.4)	26,923	(72.4)
Low income	1192	(3.2)	1192	(3.2)
ICU in medical center	11,331	(30.5)	11,331	(30.5)
Coexisting medical conditions				
Hypertension	8949	(24.1)	8949	(24.1)
Diabetes	8360	(22.5)	8360	(22.5)
Mental disorder	6725	(18.1)	6725	(18.1)
Peptic ulcer disease	6206	(16.7)	6206	(16.7)
Anemia	4507	(12.1)	4507	(12.1)
COPD	3561	(9.6)	3561	(9.6)
Fracture	2495	(6.7)	2495	(6.7)
Asthma	1250	(3.4)	1250	(3.4)
Atherosclerosis	1197	(3.2)	1197	(3.2)
Traumatic brain injury	1063	(2.9)	1063	(2.9)
Congestive heart failure	832	(2.2)	832	(2.2)
Renal dialysis	806	(2.2)	806	(2.2)
Thrombocytopenia	719	(1.9)	719	(1.9)
Reasons for ICU admission				
Digestive disease	10,198	(27.4)	10,198	(27.4)
Cancer	6726	(18.1)	6726	(18.1)
Respiratory disease	4524	(12.2)	4524	(12.2)
Circulatory disease	4385	(11.8)	4385	(11.8)
Infectious disease	3518	(9.5)	3518	(9.5)
Injury and poisoning	2918	(7.8)	2918	(7.8)
Symptom-defined conditions	862	(2.3)	862	(2.3)
Genitourinary disease	762	(2.0)	762	(2.1)
Endocrine disease	605	(1.6)	605	(1.6)
Musculoskeletal disease	598	(1.6)	598	(1.6)
Mental disorder	278	(0.8)	278	(0.8)
Neurological disease	304	(0.8)	304	(0.8)
Skin diseases	291	(0.8)	291	(0.8)
Tumor	254	(0.7)	254	(0.7)
Blood disease	76	(0.2)	76	(0.2)
Congenital anomalies	40	(0.1)	40	(0.1)
Disease of perinatal period	33	(0.1)	33	(0.1)
Pregnancy complications	7	(0.02)	7	(0.02)
Receiving surgery	15,297	(41.1)	15,297	(41.1)
Endotracheal intubation	13,076	(35.1)	13,076	(35.1)
Complications in ICU				
Septicemia	7338	(19.7)	7338	(19.7)
Pneumonia	2970	(8.0)	2970	(8.0)
Acute renal failure	1708	(4.6)	1708	(4.6)
Urinary tract infection	1681	(4.5)	1681	(4.5)
Stroke	1438	(3.9)	1438	(3.9)
Acute myocardial infarction	403	(1.1)	403	(1.1)
Pulmonary embolism	22	(0.1)	22	(0.1)

*COPD* chronic obstructive pulmonary disease, *ICU* intensive care unit

In Table 2, patients with cirrhosis showed higher ICU medical expenditure than patients without (12,008 ± 9890 vs. 11,366 ± 8742 USD, *p* < 0.0001). Cirrhosis was associated with a significant increase in 30-day mortality (aOR 1.60, 95% CI 1.53 to 1.68), ICU mortality (aOR 1.44, 95% CI 1.38 to 1.51), and one-year mortality (aOR 1.40, 95% CI 1.35 to 1.46) in ICU patients.

**Table 2:** Intensive care unit mortality in patients with and without liver cirrhosis

Outcomes of ICU	No LC, %	LC, %	OR	(95% CI)^a^
30-day mortality	10.9	15.9	1.60	(1.53 to 1.68)
ICU mortality	12.9	17.2	1.44	(1.38 to 1.51)
One-year mortality	17.3	22.1	1.40	(1.35 to 1.46)
Medical expenditure, USD^b^	11,366 ± 8742	12,008 ± 9890	*p* < 0.0001	
Length of hospital stay, days^b^	8.6 ± 29.6	8.3 ± 29.2	*p* = 0.2174	

*CI* confidence interval, *ICU* intensive care unit, *OR* odds ratio

^a^Adjusted all.covariates listed in Table 1

^b^Mean ± SD

Compared with ICU patients without cirrhosis (Table 3), cirrhotic ICU patients had increased 30-day mortality when they also had liver cancer (aOR 1.85, 95% CI 1.74 to 1.97), hepatitis B or C virus infection (aOR 1.75, 95% CI 1.50 to 2.03), alcohol dependence syndrome (aOR 1.82, 95% CI 1.62 to 2.04), jaundice (aOR 2.23, 95% CI 2.03 to 2.45), ascites (aOR 2.32, 95% CI 2.19 to 2.46), gastrointestinal hemorrhage (aOR 1.90, 95% CI 1.78 to 2.03), hepatic coma (aOR 2.21, 95% CI 2.07 to 2.36), more than 4 admissions due to LC (aOR 2.52, 95% CI 2.20 to 2.89), more than 30 days of hospital stay due to LC (aOR 2.97, 95% CI 2.66 to 3.31), and albumin supplement (aOR 1.93, 95% CI 1.83 to 2.04). The aORs of alcohol-related cirrhosis and previous hospitalization associated with 30-day mortality were 1.75 (95% CI 1.66 to 1.85) and 1.52 (95% CI 1.46 to 1.58), respectively.

**Table 3:** Stratified analysis and effects of cirrhosis-related clinical indicators on 30-day mortality of ICU patients

	n	30-day mortality			
		Deaths	Mortality, %	OR	(95% CI)^a^
Pre-ICU characteristics within 2 years					
No LC	37,197	4046	10.9	1.00	(reference)
LC without liver cancer	25,758	3705	14.4	1.48	(1.41 to 1.56)
LC with liver cancer	11,439	2190	19.2	1.85	(1.74 to 1.97)
LC with no HBV and HCV	22,258	3366	15.1	1.55	(1.47 to 1.63)
LC with HBV or HCV	13,573	2288	16.9	1.67	(1.58 to 1.77)
LC with HBV and HCV	1366	241	17.6	1.75	(1.50 to 2.03)
LC without ADS	34,496	5450	15.8	1.59	(1.52 to 1.66)
LC with ADS	2701	445	16.5	1.82	(1.62 to 2.04)
LC without jaundice	33,978	5171	15.2	1.54	(1.47 to 1.62)
LC with jaundice	3219	724	22.5	2.23	(2.03 to 2.45)
LC without ascites	26,352	3332	12.6	1.30	(1.23 to 1.37)
LC with ascites	10,845	2563	23.6	2.32	(2.19 to 2.46)
LC without GI hemorrhage	27,652	4114	14.9	1.50	(1.43 to 1.58)
LC with GI hemorrhage	9545	1781	18.7	1.90	(1.78 to 2.03)
LC without hepatic coma	29,252	4102	14.0	1.43	(1.37 to 1.37)
LC with hepatic coma	7945	1793	22.6	2.21	(2.07 to 2.36)
LC with 0 admission	25,677	3378	13.2	1.33	(1.26 to 1.40)
LC with 1 admission	6600	1411	21.4	2.16	(2.01 to 2.32)
LC with 2 admissions	2449	521	21.3	2.14	(1.92 to 2.38)
LC with 3 admissions	1059	248	23.4	2.37	(2.03 to 2.76)
LC with ≥4 admissions	1412	337	23.9	2.52	(2.20 to 2.89)
LC with 0 days of hospital stay	25,677	3378	13.2	1.33	(1.26 to 1.40)
LC with 1–9 days of hospital stay	5144	996	19.4	1.92	(1.77 to 2.08)
LC with 10–19 days of hospital stay	3022	664	22.0	2.23	(2.02 to 2.45)
LC with 20–29 days of hospital stay	1312	305	23.3	2.30	(2.00 to 2.65)
LC with ≥30 days of hospital stay	2042	552	27.0	2.97	(2.66 to 3.31)
LC without albumin supplement	23,279	3140	13.5	1.40	(1.33 to 1.47)
LC with albumin supplement	13,918	2755	19.8	1.93	(1.83 to 2.04)
LC without alcohol-related illness	24,894	3581	14.4	1.25	(1.20 to 1.31)
LC with alcohol-related illness	12,303	2314	18.8	1.75	(1.66 to 1.85)
LC without hospitalizations	7214	756	10.5	0.91	(0.84 to 0.98)
LC with hospitalizations	29,983	5139	17.1	1.52	(1.46 to 1.58)
Reasons for ICU admission					
Digestive disease, no LC	10,198	550	5.4	1.00	(reference)
Digestive disease, LC	10,198	1511	14.8	3.23	(2.91 to 3.58)
Cancer, no LC	6726	1031	15.3	1.00	(reference)
Cancer, LC	6726	1154	17.2	1.15	(1.05 to 1.27)
Respiratory disease, no LC	4524	837	18.5	1.00	(reference)
Respiratory disease, LC	4524	986	21.8	1.24	(1.12 to 1.38)
Circulatory disease, no LC	4385	384	8.8	1.00	(reference)
Circulatory disease, LC	4385	530	12.1	1.47	(1.28 to 1.70)
Infectious disease, no LC	3518	807	22.9	1.00	(reference)
Infectious disease, LC	3518	983	27.9	1.32	(1.19 to 1.48)
Injury and poisoning, no LC	2918	192	6.6	1.00	(reference)
Injury and poisoning, LC	2918	345	11.8	1.95	(1.61 to 2.35)
Symptom-defined conditions, no LC	862	123	14.3	1.00	(reference)
Symptom-defined conditions, LC	862	194	22.5	1.86	(1.43 to 2.42)
Genitourinary disease, no LC	762	38	5.0	1.00	(reference)
Genitourinary disease, LC	762	59	7.7	1.74	(1.10 to 2.75)
Endocrine disease, no LC	605	24	4.0	1.00	(reference)
Endocrine disease, LC	605	31	5.1	1.36	(0.76 to 2.43)
Musculoskeletal disease, no LC	598	17	2.8	1.00	(reference)
Musculoskeletal disease, LC	598	42	7.0	3.49	(1.79 to 6.82)
Mental disorder, no LC	278	0	0.0	1.00	(reference)
Mental disorder, LC	278	0	0.0	–	–
Neurological disease, no LC	304	11	3.6	1.00	(reference)
Neurological disease, LC	304	25	8.2	2.94	(1.30 to 6.65)
Skin disease, no LC	291	7	2.4	1.00	(reference)
Skin disease, LC	291	8	2.8	1.23	(0.35 to 4.39)
Tumor, no LC	254	2	0.8	1.00	(reference)
Tumor, LC	254	5	2.0	3.33	(0.51 to 21.8)
Blood disease, no LC	76	5	6.6	1.00	(reference)
Blood disease, LC	76	3	4.0	0.38	(0.05 to 2.84)
Congenital anomalies, no LC	40	1	2.5	1.00	(reference)
Congenital anomalies, LC	40	0	0.0	–	–
Disease of perinatal period, no LC	33	1	3.0	1.00	(reference)
Disease of perinatal period, LC	33	0	0.0	–	–
Complications of pregnancy, no LC	7	0	0.0	1.00	(reference)
Complications of pregnancy, LC	7	1	14.3	–	–
0 CCI score, no LC	10,476	1066	10.2	1.00	(reference)
0 CCI score, LC	1165	101	8.7	0.97	(0.77 to 1.23)
1 CCI score, no LC	8899	731	8.2	1.00	(reference)
1 CCI score, LC	6656	818	12.3	1.38	(1.21 to 1.58)
2 CCI score, no LC	6573	592	9.0	1.00	(reference)
2 CCI score, LC	5116	592	11.6	1.49	(1.31 to 1.70)
3 CCI score, no LC	3897	355	9.1	1.00	(reference)
3 CCI score, LC	5478	760	13.9	1.45	(1.25 to 1.69)
4 CCI score, no LC	1823	209	11.5	1.00	(reference)
4 CCI score, LC	7085	1325	18.7	1.40	(1.17 to 1.67)
≥ 5 CCI score, no LC	5529	1093	19.8	1.00	(reference)
≥ 5 CCI score, LC	11,697	2299	19.7	1.08	(0.99 to 1.18)

*ADS* alcohol dependence syndrome, *CI* confidence interval, *GI* gastrointestinal, *HBV* hepatitis B virus, *HCV* hepatitis C virus, *ICU* intensive care unit, *LC* liver cirrhosis, *OR* odds ratio

^a^Adjusted all covariates listed in Table 1

The association between liver cirrhosis and ICU mortality was significant in relation to the following causes of ICU admission: digestive disease (aOR 3.23, 95% CI 2.91 to 3.58), cancer (aOR 1.15, 95% CI 1.05 to 1.27), respiratory disease (aOR 1.24, 95% CI 1.12 to 1.38), circulatory disease (aOR 1.47, 95% CI 1.28 to 1.70), infectious disease (aOR 1.32, 95% CI 1.19 to 1.48), injury and poisoning (aOR 1.95, 95% CI 1.61 to 2.35), symptom-defined conditions (aOR 1.86, 95% CI 1.43 to 2.42), genitourinary disease (aOR 1.74, 95% CI 1.10 to 2.75), musculoskeletal disease (aOR 3.49, 95% CI 1.79 to 6.82), and neurological disease (aOR 2.94, 95% CI 1.30 to 6.65). The 30-day mortality was also associated with LC in ICU patients with 2 scores (aOR 1.49, 95% CI 1.31 to 1.70), 3 scores (aOR 1.45, 95% CI 1.25 to 1.69), and 4 scores (aOR 1.40, 95% CI 1.17 to 1.67) of Charlson Comorbidity Index.

Stratified analysis and effects of cirrhosis-related clinical indicators on ICU mortality and one-year mortality of ICU patients were showed in Additional file 1: Tables S2 and S3. The actual survival starting at the day of ICU admission in patients with and without liver cirrhosis was showed in Additional file 1: Table S4.

## Discussion

In this large-scale, nationwide, population-based propensity score-matched study, patients with cirrhosis of the liver admitted to ICU showed significantly higher ICU mortality as well as increased medical expenditure compared with non-cirrhotic controls. The stratified analyses showed higher ICU mortality among patients with increasing numbers of co-morbidities. Associated with even higher ICU mortality were cirrhosis-related clinical conditions, liver cancer, alcohol dependence syndrome, jaundice, ascites, gastrointestinal hemorrhage and hepatic coma. Regarding long-term outcomes after ICU discharge, higher one-year mortality was noted in liver cirrhosis patients with older age, male gender, ICU in medical center, anemia, renal dialysis, congestive heart failure, and complications in ICU such as septicemia and pneumonia.

To the best of our knowledge, this is the first report investigating the influence of liver cirrhosis on 30-day mortality, ICU mortality, and one-year mortality in ICU patients using a nationwide database. Previous studies were mostly conducted from a single center [10, 12, 21–24], which may represent a certain type of patient group and medical practice. Although these studies evaluated risk factors among patients with liver cirrhosis admitted to ICU, they did not note various cirrhosis-related clinical characteristics nor assess the impact of cirrhosis per se, adjusting all covariates with the control group to the ICU mortality [7, 10–12, 25, 26]. In our subgroup analyses, the odds ratios of ICU mortality in cirrhotic patients’ ICU admissions with primary diagnoses such as cancer, respiratory disease and infectious disease were lower than other causes of ICU admission. This might be attributed to cancer, pneumonia, COPD and sepsis having more impact on ICU mortality than cirrhosis after adjustment [27–29].

Regarding the effects of cirrhosis-related clinical indicators on ICU mortality, cirrhotic patients with liver cancer, alcohol dependence syndrome, jaundice, ascites, gastrointestinal hemorrhage and hepatic coma had higher ICU mortality than those without cirrhosis. Of these cirrhosis-related clinical indicators, jaundice, ascites and hepatic coma were consistent with the Child-Pugh score risk factors of the most commonly used prognosis predicting model in cirrhotic patients [13]. The development of ascites, gastrointestinal hemorrhage, encephalopathy and jaundice mark the decompensated stage of liver cirrhosis that results in poorer prognoses [30, 31]. These complications might contribute to the increased ICU mortality found in patients of liver cirrhosis with more hospitalization. Liver cancer, especially hepatocellular carcinoma, is one of the leading causes of death in Taiwan [32], so it is reasonable that cirrhotic patients with liver cancer would have higher ICU mortality than those without it.

Regarding increased ICU mortality and medical expenditures, there are some possible explanations why patients with liver cirrhosis had worse outcomes. First, patients with liver cirrhosis are presumed to have impaired immune function and are thus more susceptible to severe infection, leading to higher mortality [33, 34]. A previous study demonstrated that infection increased mortality in patients with cirrhosis of liver fourfold, with 30% of patients dying within a month of infection and another 30% dying within a year [35]. This was consistent with our finding that septicemia and pneumonia in patients with cirrhosis were associated with higher ICU mortality. Second, portal hypertension and subsequent esophageal variceal bleeding and ascites play major roles as complications of cirrhosis and are associated with 1-year mortality of nearly 20% [1, 5, 36]. Third, as cirrhosis progresses, the development of renal vasoconstriction leads to hepatorenal syndrome. Renal failure is an indicator of end-stage liver disease and increases mortality risk by seven times, with half of patients dying within a month [37]. In our study, acute renal failure was also significantly associated with ICU mortality. To reduce ICU mortality in patients with liver cirrhosis, health care teams should optimize management of these specific issues according to updated guidelines.

Concerning long-term outcomes, variables such as serum albumin or bilirubin levels, ascites, encephalopathy, and prothrombin time for the Child-Pugh score are the most common independent predictors of mortality in patients with liver cirrhosis [5]. In our national cohort, ICU mortality among patients with liver cirrhosis increased with numbers of cirrhosis-related clinical conditions. These findings were compatible with previous studies demonstrating high mortality in cirrhotic patients with renal failure and gastrointestinal hemorrhage [37, 38]. However, our study focused on impacts on long-term mortality after ICU discharge, which was not investigated before. Considering specific management of these factors for ICU patients with cirrhosis of the liver is warranted to reduce mortality.

The present study has strengths of large sample sizes and adjustment for potential confounding factors by propensity score-matching method in a nationwide population-based retrospective cohort. It also has some limitations encountered in research based on secondary data. First, detailed information on laboratory data, physical examinations, and hemodynamic parameters was not available from reimbursement claim data. For example, the international normalized ratio of prothrombin time, bilirubin and creatinine level in blood would help to predict outcomes among cirrhosis patients [13, 14]. Second, severity of liver cirrhosis noted by Child-Pugh score, Model For End-Stage Liver Disease score or other criteria was not found in reimbursement data for risk stratification of ICU mortality. Third, though the accuracy of major diagnosis codes from the Taiwan National Health Insurance Research Database has been accepted by scientific journals [17–20], the validity of liver cirrhosis, comorbidity and complication codes employed in this study might still be questioned. To reduce the possibility of misdiagnosis or miscoding, we applied inclusive criteria of at least two visits for medical services with physician’s primary diagnosis of liver cirrhosis. In addition, an important factor influencing ICU outcome is Do Not Resuscitate orders. However, we have no data regarding the Do Not Resuscitate order in this study because of the limitations of Taiwan’s National Health Insurance Research Database. Finally, we could not exclude the possibility that some patients with hepatitis without cirrhosis were included in cirrhotic group in this study because the diagnosis error by physicians may occur in the clinical settings.

## Conclusions

In conclusion, this nationwide population-based study showed that patients with liver cirrhosis admitted to ICU have higher ICU and one-year mortality after discharge in patterns that closely correlate with medical conditions and specific scenarios. These findings can help health care providers develop specific protocols to improve prognosis and long-term survival rates for ICU patients with liver cirrhosis.

## Supplementary information

**Additional file 1: Figure S1.** Flow chart for selecting intensive care unit patients with or without liver cirrhosis, 2006-2013. **Table S1.** International Classification of Diseases, Ninth Revision, Clinical Modification (ICD-9-CM) codes of diseases. **Table S2.** Stratified analysis and effects of cirrhosis-related clinical indicators on ICU mortality. **Table S3.** Stratified analysis and effects of cirrhosis-related clinical indicators on one-year mortality of ICU patients. **Table S4.** The actual survival starting at the day of ICU admission in patients with and without liver cirrhosis

## Data Availability

Our data will not be shared because of the regulations from the Health and Welfare Data Science Center (HWDC). The data underlying this study is from the National Health Insurance Research Database (NHIRD), which has been transferred to the HWDC. Interested researchers can obtain the data through formal application to the HWDC, Department of Statistics, Ministry of Health and Welfare, Taiwan (http://dep.mohw.gov.tw/DOS/np-2497-113.html).

## Abbreviations

CI: Confidence interval
ICD-9-CM: *International Classification of Diseases, 9th Revision, Clinical Modification*
ICU: Intensive care unit
OR: Odds ratio
