# Supplementary material for: A population-based cohort study of mortality of intensive care unit patients with liver cirrhosis
Source: BMC Gastroenterol. 2020 Jan 16;20:15. doi: 10.1186/s12876-020-1163-1 (PMC6966823; doi:10.1186/s12876-020-1163-1)
Supplement: Supplementary file 1 — Additional file 1: Figure S1. Flow chart for selecting intensive care unit patients with or without liver cirrhosis, 2006-2013. Table S1. International Classification of Diseases, Ninth Revision, Clinical Modification (ICD-9-CM) codes of diseases. Table S2. Stratified analysis and effects of cirrhosis-related clinical indicators on ICU mortality. Table S3. Stratified analysis and effects of cirrhosis-related clinical indicators on one-year mortality of ICU patients. Table S4. The actual survival starting at the day of ICU admission in patients with and without liver cirrhosis [file 12876_2020_1163_MOESM1_ESM.doc]

**Figure S1.** Flow chart for selecting intensive care unit patients with or without liver cirrhosis, 2006-2013.


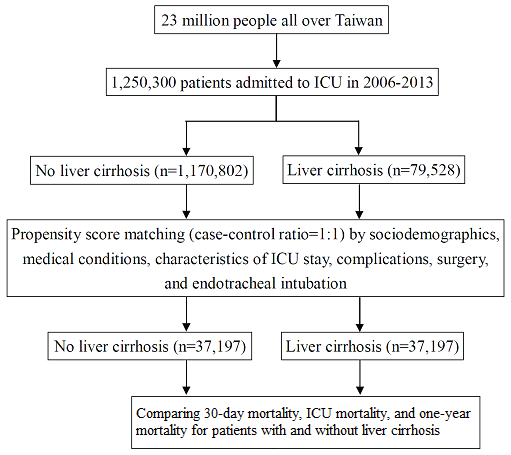


| **Table S1** International Classification of Diseases, Ninth Revision, Clinical Modification (ICD-9-CM) codes of diseases | |
| --- | --- |
| Disease | ICD-9-CM code |
| Liver cirrhosis | 571.2, 571.5, 571.6 |
| Diabetes | 250 |
| Hypertension | 401-405 |
| Mental disorders | 290-319 |
| Chronic obstructive pulmonary disease | 490-496 |
| Fracture | 800-829 |
| Pneumonia | 480-486 |
| Stroke | 430-438 |
| Asthma | 493 |
| Traumatic brain injury | 850-854 |
| Congestive heart failure | 428 |
| Immune thrombocytopenia | 287 |
| Hyperlipidemia | 272.0, 272.1, 272.2 |
| Epilepsy | 345 |
| Atrial fibrillation | 427.3 |
| Peripheral vascular disease | 443 |
| Systemic lupus erythematosus | 710.0 |
| Renal dialysis | administration code D8, D9 |
| Septicemia | 038, 998.5 |
| Acute renal failure | 584 |
| Urinary tract infection | 599.0 |
| Acute myocardial infarction | 410 |
| Pulmonary embolism | 415 |
| Digestive disease | 520-579 |
| Cancer | 140-209, 230-234 |
| Respiratory disease | 460-519 |
| Circulatory disease | 390-459 |
| Infectious disease | 001-139 |
| Injury and poisoning | 800-999 |
| Symptom-defined conditions | 780-799 |
| Genitourinary disease | 580-629 |
| Endocrine disease | 240-279 |
| Musculoskeletal disease | 710-739 |
| Neurological disease | 320-389 |
| Skin disease | 680-709 |
| Tumor | 210-229, 235-239 |
| Blood disease | 280-289 |
| Congenital anomalies | 740-759 |
| Disease of perinatal period | 760-779 |
| Pregnancy complications | 630-677 |
| ICD-9-CM=International Classification of Diseases, Ninth Revision, Clinical Modification. | |

| **Table S2** Stratified analysis and effects of cirrhosis-related clinical indicators on ICU mortality | | | | | |
| --- | --- | --- | --- | --- | --- |
|  |  | ICU mortality | | | |
|  | n | Deaths | Mortality, % | OR | (95% CI)^a^ |
| Pre-ICU characteristics within 2 years |  |  |  |  |  |
| No LC | 37197 | 4814 | 12.9 | 1.00 | (reference) |
| LC without liver cancer | 25758 | 4153 | 16.1 | 1.41 | (1.34 to 1.48) |
| LC with liver cancer | 11439 | 2231 | 19.5 | 1.51 | (1.42 to 1.60) |
| LC with no HBV and HCV | 22258 | 3709 | 16.7 | 1.44 | (1.37 to 1.51) |
| LC with HBV or HCV | 13573 | 2426 | 17.9 | 1.45 | (1.37 to 1.53) |
| LC with HBV and HCV | 1366 | 249 | 18.2 | 1.46 | (1.26 to 1.69) |
| LC without ADS | 34496 | 5889 | 17.1 | 1.42 | (1.36 to 1.49) |
| LC with ADS | 2701 | 495 | 18.3 | 1.72 | (1.54 to 1.93) |
| LC without jaundice | 33978 | 5615 | 16.5 | 1.39 | (1.33 to 1.46) |
| LC with jaundice | 3219 | 769 | 23.9 | 1.96 | (1.79 to 2.15) |
| LC without ascites | 26352 | 3666 | 13.9 | 1.19 | (1.13 to 1.25) |
| LC with ascites | 10845 | 2718 | 25.1 | 2.03 | (1.92 to 2.15) |
| LC without GI hemorrhage | 27652 | 4474 | 16.2 | 1.36 | (1.30 to 1.42) |
| LC with GI hemorrhage | 9545 | 1910 | 20.0 | 1.69 | (1.59 to 1.80) |
| LC without hepatic coma | 29252 | 4442 | 15.2 | 1.29 | (1.23 to 1.35) |
| LC with hepatic coma | 7945 | 1942 | 24.4 | 2.00 | (1.88 to 2.13) |
| LC with 0 admission | 25677 | 3684 | 14.4 | 1.21 | (1.15 to 1.27) |
| LC with 1 admission | 6600 | 1525 | 23.1 | 1.94 | (1.81 to 2.08) |
| LC with 2 admissions | 2449 | 555 | 22.7 | 1.89 | (1.70 to 2.10) |
| LC with 3 admissions | 1059 | 261 | 24.7 | 2.06 | (1.77 to 2.39) |
| LC with ≥4 admissions | 1412 | 359 | 25.4 | 2.24 | (1.96 to 2.56) |
| LC with 0 days of hospital stay | 25677 | 3684 | 14.4 | 1.20 | (1.15 to 1.27) |
| LC with 1-9 days of hospital stay | 5144 | 1059 | 20.6 | 1.69 | (1.56 to 1.83) |
| LC with 10-19 days of hospital stay | 3022 | 717 | 23.7 | 2.01 | (1.83 to 2.21) |
| LC with 20-29 days of hospital stay | 1312 | 316 | 24.1 | 1.95 | (1.69 to 2.23) |
| LC with ≥30 days of hospital stay | 2042 | 608 | 29.8 | 2.77 | (2.49 to 3.08) |
| LC without albumin supplement | 23279 | 3416 | 14.7 | 1.27 | (1.21 to 1.33) |
| LC with albumin supplement | 13918 | 2968 | 21.3 | 1.72 | (1.63 to 1.81) |
| Reasons for ICU admission |  |  |  |  |  |
| Digestive disease, no LC | 10198 | 725 | 7.1 | 1.00 | (reference) |
| Digestive disease, LC | 10198 | 1689 | 16.6 | 2.76 | (2.51 to 3.04) |
| Cancer, no LC | 6726 | 1236 | 18.4 | 1.00 | (reference) |
| Cancer, LC | 6726 | 1210 | 18.0 | 0.97 | (0.89 to 1.07) |
| Respiratory disease, no LC | 4524 | 1005 | 22.2 | 1.00 | (reference) |
| Respiratory disease, LC | 4524 | 1106 | 24.5 | 1.14 | (1.03 to 1.26) |
| Circulatory disease, no LC | 4385 | 418 | 9.5 | 1.00 | (reference) |
| Circulatory disease, LC | 4385 | 546 | 12.5 | 1.39 | (1.20 to 1.59) |
| Infectious disease, no LC | 3518 | 923 | 26.2 | 1.00 | (reference) |
| Infectious disease, LC | 3518 | 1050 | 29.9 | 1.21 | (1.09 to 1.35) |
| Injury and poisoning, no LC | 2918 | 212 | 7.3 | 1.00 | (reference) |
| Injury and poisoning, LC | 2918 | 361 | 12.4 | 1.85 | (1.54 to 2.22) |
| Symptom-defined conditions, no LC | 862 | 132 | 15.3 | 1.00 | (reference) |
| Symptom-defined conditions, LC | 862 | 197 | 22.9 | 1.73 | (1.34 to 2.24) |
| Genitourinary disease, no LC | 762 | 49 | 6.4 | 1.00 | (reference) |
| Genitourinary disease, LC | 762 | 70 | 9.2 | 1.60 | (1.05 to 2.44) |
| Endocrine disease, no LC | 605 | 26 | 4.3 | 1.00 | (reference) |
| Endocrine disease, LC | 605 | 28 | 4.6 | 1.09 | (0.61 to 1.96) |
| Musculoskeletal disease, no LC | 598 | 26 | 4.4 | 1.00 | (reference) |
| Musculoskeletal disease, LC | 598 | 47 | 7.9 | 2.29 | (1.29 to 4.05) |
| Mental disorder, no LC | 278 | 1 | 0.4 | 1.00 | (reference) |
| Mental disorder, LC | 278 | 2 | 0.7 | 2.26 | (0.17 to 30.5) |
| Neurological disease, no LC | 304 | 13 | 4.3 | 1.00 | (reference) |
| Neurological disease, LC | 304 | 26 | 8.6 | 2.47 | (2.47 to 5.31) |
| Skin disease, no LC | 291 | 11 | 3.8 | 1.00 | (reference) |
| Skin disease, LC | 291 | 13 | 4.5 | 1.24 | (0.50 to 3.08) |
| Tumor, no LC | 254 | 4 | 1.6 | 1.00 | (reference) |
| Tumor, LC | 254 | 4 | 1.6 | 1.00 | (0.21 to 4.67) |
| Blood disease, no LC | 76 | 4 | 5.3 | 1.00 | (reference) |
| Blood disease, LC | 76 | 4 | 5.3 | 1.00 | (0.14 to 7.13) |
| Congenital anomalies, no LC | 40 | 2 | 5.0 | 1.00 | (reference) |
| Congenital anomalies, LC | 40 | 1 | 2.5 | 0.25 | (0.01 to 7.44) |
| Disease of perinatal period, no LC | 33 | 1 | 3.0 | 1.00 | (reference) |
| Disease of perinatal period, LC | 33 | 0 | 0.0 | - | - |
| Complications of pregnancy, no LC | 7 | 0 | 0.0 | 1.00 | (reference) |
| Complications of pregnancy, LC | 7 | 0 | 0.0 | - | - |
| 0 CCI score, no LC | 10476 | 1249 | 11.9 | 1.00 | (reference) |
| 0 CCI score, LC | 1165 | 112 | 9.6 | 0.92 | (0.74 to 1.15) |
| 1 CCI score, no LC | 8899 | 879 | 9.9 | 1.00 | (reference) |
| 1 CCI score, LC | 6656 | 918 | 13.8 | 1.28 | (1.13 to 1.45) |
| 2 CCI score, no LC | 6573 | 759 | 11.6 | 1.00 | (reference) |
| 2 CCI score, LC | 5116 | 669 | 13.1 | 1.32 | (1.16 to 1.49) |
| 3 CCI score, no LC | 3897 | 440 | 11.3 | 1.00 | (reference) |
| 3 CCI score, LC | 5478 | 801 | 14.6 | 1.22 | (1.06 to 1.40) |
| 4 CCI score, no LC | 1823 | 260 | 14.3 | 1.00 | (reference) |
| 4 CCI score, LC | 7085 | 1462 | 20.6 | 1.25 | (1.06 to 1.47) |
| ≥5 CCI score, no LC | 5529 | 1227 | 22.2 | 1.00 | (reference) |
| ≥5 CCI score, LC | 11697 | 2422 | 20.7 | 0.98 | (0.90 to 1.07) |
| ADS = alcohol dependence syndrome; CI = confidence interval; GI = gastrointestinal; HBV = hepatitis B virus; HCV = hepatitis C virus; ICU = intensive care unit; LC = liver cirrhosis; OR = odds ratio.  ^a^Adjusted for all covariates listed in Table 1. | | | | | |

| **Table S3** Stratified analysis and effects of cirrhosis-related clinical indicators on one-year mortality of ICU patients | | | | | |
| --- | --- | --- | --- | --- | --- |
|  |  | One-year mortality | | | |
|  | n | Deaths | Mortality, % | OR | (95% CI)^a^ |
| Pre-ICU characteristics within 2 years |  |  |  |  |  |
| No LC | 37197 | 6426 | 17.3 | 1.00 | (reference) |
| LC without liver cancer | 25758 | 5128 | 19.9 | 1.35 | (1.29 to 1.41) |
| LC with liver cancer | 11439 | 3100 | 27.1 | 1.51 | (1.43 to 1.59) |
| LC with no HBV and HCV | 22258 | 4650 | 20.9 | 1.38 | (1.32 to 1.44) |
| LC with HBV or HCV | 13573 | 3233 | 23.8 | 1.43 | (1.36 to 1.51) |
| LC with HBV and HCV | 1366 | 345 | 25.3 | 1.52 | (1.33 to 1.73) |
| LC without ADS | 34496 | 7622 | 22.1 | 1.38 | (1.33 to 1.44) |
| LC with ADS | 2701 | 606 | 22.4 | 1.69 | (1.52 to 1.87) |
| LC without jaundice | 33978 | 7285 | 21.4 | 1.35 | (1.30 to 1.41) |
| LC with jaundice | 3219 | 943 | 29.3 | 1.95 | (1.79 to 2.12) |
| LC without ascites | 26352 | 4818 | 18.3 | 1.16 | (1.11 to 1.21) |
| LC with ascites | 10845 | 3410 | 31.4 | 2.02 | (1.92 to 2.13) |
| LC without GI hemorrhage | 27652 | 5709 | 20.7 | 1.30 | (1.24 to 1.35) |
| LC with GI hemorrhage | 9545 | 2519 | 26.4 | 1.72 | (1.63 to 1.82) |
| LC without hepatic coma | 29252 | 5802 | 19.8 | 1.25 | (1.20 to 1.30) |
| LC with hepatic coma | 7945 | 2426 | 30.5 | 2.00 | (1.89 to 2.12) |
| LC with 0 admission | 25677 | 4862 | 18.9 | 1.18 | (1.13 to 1.23) |
| LC with 1 admission | 6600 | 1873 | 28.4 | 1.85 | (1.74 to 1.98) |
| LC with 2 admissions | 2449 | 693 | 28.3 | 1.87 | (1.70 to 2.06) |
| LC with 3 admissions | 1059 | 329 | 31.1 | 2.10 | (1.82 to 2.42) |
| LC with ≥4 admissions | 1412 | 471 | 33.4 | 2.46 | (2.18 to 2.78) |
| LC with 0 days of hospital stay | 25677 | 4862 | 18.9 | 1.18 | (1.13 to 1.23) |
| LC with 1-9 days of hospital stay | 5144 | 1315 | 25.6 | 1.62 | (1.51 to 1.74) |
| LC with 10-19 days of hospital stay | 3022 | 889 | 29.4 | 1.96 | (1.80 to 2.14) |
| LC with 20-29 days of hospital stay | 1312 | 409 | 31.2 | 2.07 | (1.82 to 2.35) |
| LC with ≥30 days of hospital stay | 2042 | 753 | 36.9 | 2.82 | (2.55 to 3.12) |
| LC without albumin supplement | 23279 | 4370 | 18.8 | 1.22 | (1.17 to 1.28) |
| LC with albumin supplement | 13918 | 3858 | 27.7 | 1.70 | (1.62 to 1.78) |
| Reasons for ICU admission |  |  |  |  |  |
| Digestive disease, no LC | 10198 | 872 | 8.6 | 1.00 | (reference) |
| Digestive disease, LC | 10198 | 2153 | 21.1 | 3.03 | (2.78 to 3.30) |
| Cancer, no LC | 6726 | 2009 | 29.9 | 1.00 | (reference) |
| Cancer, LC | 6726 | 1791 | 26.6 | 0.84 | (0.78 to 0.91) |
| Respiratory disease, no LC | 4524 | 1300 | 28.7 | 1.00 | (reference) |
| Respiratory disease, LC | 4524 | 1382 | 30.6 | 1.10 | (1.00 to 1.20) |
| Circulatory disease, no LC | 4385 | 492 | 11.2 | 1.00 | (reference) |
| Circulatory disease, LC | 4385 | 664 | 15.1 | 1.46 | (1.28 to 1.66) |
| Infectious disease, no LC | 3518 | 1079 | 30.7 | 1.00 | (reference) |
| Infectious disease, LC | 3518 | 1188 | 33.8 | 1.16 | (1.05 to 1.29) |
| Injury and poisoning, no LC | 2918 | 230 | 7.9 | 1.00 | (reference) |
| Injury and poisoning, LC | 2918 | 421 | 14.4 | 2.03 | (1.71 to 2.41) |
| Symptom-defined conditions, no LC | 862 | 155 | 18.0 | 1.00 | (reference) |
| Symptom-defined conditions, LC | 862 | 224 | 26.0 | 1.69 | (1.32 to 2.15) |
| Genitourinary disease, no LC | 762 | 76 | 10.0 | 1.00 | (reference) |
| Genitourinary disease, LC | 762 | 115 | 15.1 | 1.73 | (1.24 to 2.41) |
| Endocrine disease, no LC | 605 | 42 | 6.9 | 1.00 | (reference) |
| Endocrine disease, LC | 605 | 52 | 8.6 | 1.28 | (0.83 to 1.99) |
| Musculoskeletal disease, no LC | 598 | 29 | 4.9 | 1.00 | (reference) |
| Musculoskeletal disease, LC | 598 | 53 | 8.9 | 2.30 | (1.35 to 3.94) |
| Mental disorder, no LC | 278 | 0 | 0.0 | 1.00 | (reference) |
| Mental disorder, LC | 278 | 4 | 1.4 | - | - |
| Neurological disease, no LC | 304 | 16 | 5.3 | 1.00 | (reference) |
| Neurological disease, LC | 304 | 37 | 12.2 | 2.96 | (1.51 to 5.78) |
| Skin disease, no LC | 291 | 10 | 3.4 | 1.00 | (reference) |
| Skin disease, LC | 291 | 18 | 6.2 | 2.04 | (0.87 to 4.80) |
| Tumor, no LC | 254 | 5 | 2.0 | 1.00 | (reference) |
| Tumor, LC | 254 | 7 | 2.8 | 1.49 | (0.43 to 5.21) |
| Blood disease, no LC | 76 | 8 | 10.5 | 1.00 | (reference) |
| Blood disease, LC | 76 | 10 | 13.2 | 1.36 | (0.46 to 4.06) |
| Congenital anomalies, no LC | 40 | 1 | 2.5 | 1.00 | (reference) |
| Congenital anomalies, LC | 40 | 1 | 2.5 | 1.00 | (0.02 to 50.1) |
| Disease of perinatal period, no LC | 33 | 1 | 3.0 | 1.00 | (reference) |
| Disease of perinatal period, LC | 33 | 0 | 0.0 | - | - |
| Complications of pregnancy, no LC | 7 | 0 | 0.0 | 1.00 | (reference) |
| Complications of pregnancy, LC | 7 | 1 | 14.3 | - | - |
| 0 CCI score, no LC | 10476 | 1415 | 13.5 | 1.00 | (reference) |
| 0 CCI score, LC | 1165 | 133 | 11.4 | 0.94 | (0.76 to 1.15) |
| 1 CCI score, no LC | 8899 | 1082 | 12.2 | 1.00 | (reference) |
| 1 CCI score, LC | 6656 | 1080 | 16.2 | 1.28 | (1.14 to 1.43) |
| 2 CCI score, no LC | 6573 | 1022 | 15.6 | 1.00 | (reference) |
| 2 CCI score, LC | 5116 | 850 | 16.6 | 1.28 | (1.15 to 1.43) |
| 3 CCI score, no LC | 3897 | 606 | 15.6 | 1.00 | (reference) |
| 3 CCI score, LC | 5478 | 1093 | 20.0 | 1.23 | (1.08 to 1.39) |
| 4 CCI score, no LC | 1823 | 355 | 19.5 | 1.00 | (reference) |
| 4 CCI score, LC | 7085 | 1772 | 25.0 | 1.17 | (1.01 to 1.36) |
| ≥5 CCI score, no LC | 5529 | 1946 | 35.2 | 1.00 | (reference) |
| ≥5 CCI score, LC | 11697 | 3300 | 28.2 | 0.83 | (0.77 to 0.90) |
| ADS = alcohol dependence syndrome; CI = confidence interval; GI = gastrointestinal; HBV = hepatitis B virus; HCV = hepatitis C virus; ICU = intensive care unit; LC = liver cirrhosis; OR = odds ratio.  ^a^Adjusted for all covariates listed in Table 1. | | | | | |

| **Table S4** The actual survival starting at the day of ICU admission in patients with and without liver cirrhosis | | | | | | |
| --- | --- | --- | --- | --- | --- | --- |
|  | No LC | | LC | | Risk of mortality | |
|  | n | % | n | % | OR | (95% CI)^a^ |
| 30-day mortality | 4046 | 10.9 | 5895 | 15.9 | 1.60 | (1.53-1.68) |
| 60- day mortality | 5043 | 13.6 | 6935 | 18.6 | 1.52 | (1.46-1.58) |
| 90- day mortality | 5459 | 14.7 | 7302 | 19.6 | 1.47 | (1.42-1.54) |
| 180-day mortality | 5994 | 16.1 | 7751 | 20.8 | 1.42 | (1.36-1.47) |
| One-year mortality | 6426 | 17.3 | 8228 | 22.1 | 1.40 | (1.35-1.46) |
| CI = confidence interval; ICU = intensive care unit; LC = liver cirrhosis; OR = odds ratio.  ^a^Adjusted for all covariates listed in Table 1. | | | | | | |
